# Acidic Phospholipase A2-Peptide Derivative Modulates Oxidative Status and Microstructural Reorganization of Scar Tissue after Cutaneous Injury

**DOI:** 10.1155/2020/8273986

**Published:** 2020-07-11

**Authors:** Estefanny Ruiz García, Edvaldo Barros, Stephanie Stransky, Carlos Chávez-Olórtegui, Mariella Bontempo Freitas, Rômulo Dias Novaes, Reggiani Vilela Gonçalves

**Affiliations:** ^1^Department of Animal Biology, Federal University of Viçosa, Viçosa, MG, Brazil; ^2^Biomolecules Nucleus, Federal University of Viçosa, Viçosa, MG, Brazil; ^3^Department of Biochemistry and Immunology, Federal University of Minas Gerais, Belo Horizonte, MG, Brazil; ^4^Institute of Biomedical Science, Department of Structural Biology, Federal University of Alfenas, Alfenas 37130-001, MG, Brazil

## Abstract

From in vitro and in vivo models, the proliferative and healing potential of an acidic phospholipase A2 (LAPLA2) from *Lachesis muta* venom was investigated. The LAPLA2 proliferative activity was evaluated on fibroblasts and keratinocytes cultured, and the antioxidant and regenerative potential of LAPLA2 was analyzed in a murine model. The animal study consisted of four groups: C (negative control): 0.9% NaCl; SS (positive control): 1% silver sulfadiazine; L1 group: 0.5% LAPLA2; and L2 group: 0.25% LAPLA2. Wounds were topically treated daily for 12 days, and scar tissue samples were collected every 4 days. In vitro, LAPLA2 stimulated marked time-dependent cell proliferation. In vivo, it increased the antioxidant activity of superoxide dismutase (SOD) and catalase (CAT) and decreased malondialdehyde (MDA) and carbonyl protein (CP) levels in scar tissue treated with LAPLA2 at 0.5%. This peptide was effective in stimulating cellular proliferation, neoangiogenesis, type I and III collagen deposition, and maturation in a time-dependent-way, reducing the time required for wound closure. Our results indicated that LAPLA2 presented a remarkable potential in improving the oxidative status and microstructural reorganization of the scar tissue by stimulation of cellularity, angiogenesis, colagenogenesis, and wound contraction, suggesting that the peptide could be a potential candidate for a new healing drug.

## 1. Introduction

Due to complexity and interdependence of all phases of wound healing (hemostasis, inflammation, proliferation, and remodeling), disturbances in tissue repair are not uncommon, especially when associated to metabolic (e.g., diabetes mellitus and vasculopathies) and microbial diseases (e.g., bacterial, fungal, and parasitic infections). As innate and acquired immunological mechanisms are integrated in the skin to provide microbiological protection, cutaneous wounds increase the risk of opportunistic infections, sepsis, and death [1, 2]. Often disguised as a comorbidity, chronic wounds represent a silent epidemic, whose treatment is expensive, time-consuming, and not always successful [3, 4]. Only in the United States of America, at least 28.1 billion dollars is spent annually to treat all categories of wounds [5]. In this sense, the high demand for more efficient treatments for skin wounds has stimulated the discovery and inclusion of synthetic and natural products in several research and development platforms [6–10].

Although poorly explored, animal bioactive substances have aroused growing biotechnological interest for the development of more effective healing drugs, especially due to their potent antimicrobial effects and high potential for stimulating cell proliferation and collagen biosynthesis [11, 12]. Although small peptides secreted by honey bees [13] and anurans [14] are effective in accelerating the wound healing, little is known about whether and to what extent snake venom could be a source of bioactive products with potential applicability in regenerative medicine [15]. Currently, there is evidence, although limited, indicating that the antibacterial and antioxidant potential of proteins isolated from snake venom (e.g., disintegrins and phospholipases) are associated to their healing properties [6, 11, 16]. However, the impact of snake venom-isolated peptides on fibroblasts and keratinocytes proliferation, as well as collagen dynamics remains poorly understood, making it difficult to clarify its healing potential in skin wounds.

Transcriptional studies of the venom produced by the snake species *Lachesis muta muta* revealed a wide variety of protein families, such as metalloproteinases, phospholipases A2 (PLA2s), serine proteases, C-type lectin, bradykinin-potentiating peptides (BPPs), and L-amino acid oxidases (LAAOs), all with potential biotechnological and pharmacological applicabilities [17]. PLA2 covers 8.7% of proteins in *L. m. muta* venom, and its hemolytic, neurotoxic, myotoxic, and proliferative effects have been described [18, 19]. PLA2 superfamily includes five types of enzymes such as cytosolic, secreted, calcium-independent, lysosomal, and platelet-activating factors [20]. Snake venom PLA2s have been classified in the group I (in elapids) or II (in viperids) [21]. The PLA2s II are divided into two types, acidic Asp49–PLA2s, with the catalytic active activity and basic PLA2 homologues, and PLA2–like proteins that are catalytically inactive and usually possess a Lys49 substitution [22, 23]. Most of the acidic isoforms do not present toxic effects, a desirable characteristic considering its biological safety and pharmacological relevance [24].

In order to address the need for more effective treatments, and considering that *L. m. muta* venom is a viable source rich in bioactive peptides with antimicrobial, immunomodulatory, and healing potential, we isolated an acidic phospholipase A2 (LAPLA2) produced by this species and used in vitro- and in vivo-integrated approaches to investigate the effect of this molecule on fibroblasts and keratinocytes proliferation, angiogenesis, colagenogenesis, antioxidant defenses, and wound closure in a murine excisional wound model. We hypothesized that snake venom may be a valuable source of bioactive molecules with potential applicability in regenerative medicine.

## 2. Material and Methods

### 2.1. Venom, Purified Fraction

Crude venom of *L. m. muta* and its purified fraction were donated by the Immunochemistry of Proteins Laboratory of the Federal University of Minas Gerais (Belo Horizonte, Brazil). Briefly, for molecular exclusion chromatography, *L. m. muta* venom was purified according to the method used by Flores-Sánchez et al. [25]. The venom was applied to a Superdex^™^ 75 HR10/30 column (Pharmacia), previously, equilibrated with 50 mM ammonium acetate buffer, pH 6. Elution was carried out using the same buffer. Fractions were monitored through spectrophotometry at 280 nm. Samples were stored at 4–20°C until its use (unpublished results).

### 2.2. Electrophoresis and Mass Spectrometry

Protein quantification in the purified venom was performed according to Lowry [26]. In order to estimate the molecular weight of the proteins, 40 *μ*g of venom fraction was analyzed using SDS-PAGE (15%) following the method of Laemmli [27]. Protein samples were analyzed under reducing conditions. The gel was stained with colloidal Coomassie Blue G-250 (Sigma Aldrich, St. Louis, MO, USA).

The banding pattern visualized in the SDS-PAGE was subjected to trypsinization according to the protocol described by Olsen [28]. Peptide mass spectra (MS and MS/MS) were obtained using MALDI-TOF/TOF. The MALDI analysis was performed using an *α*-cyano-4-hydroxycinnamic matrix and an Ultraflex III MALDI-TOF/TOF mass spectrometer (matrix-assisted laser desorption/ionization time-of-flight) (Bruker Daltonics, Bremen, Germany). The MS analysis was performed with reflective positive mode, while the MS/MS analysis used the LIFT positive mode.

### 2.3. Spectrum Analysis and Protein Identification

Protein identification was performed using MASCOT DAEMON 2.2 software (Matrix Science, Boston, MA, USA) in the MS/MS ion search mode, following digestion with trypsin, accepting one missed cleavage, and adopting a mass error tolerance of 0.2 Da for the MS mode and 0.5 Da for the MS/MS mode. Cysteine carbamidomethylation was used as the fixed modification and methionine oxidation as the variable. The database used was Metazoa (downloaded in 09/22/2016, with 8,585,752 entries), deposited in UniProt consortium. Protein identification validation was performed using the software MASCOT (SCAFFOLD) version 3.6.4 (Proteome Software, Portland, OR, USA). For validation, the following parameters were accepted: peptides identification score ≥90% for both peptides [29] and proteins [30], with at least two validated peptides per protein. Four available sequences corresponding to acidic Asp49-PLAs were characterized from the Viperidae family, with an identity of at least 34% in comparison to the partial sequence of the purified protein, using a BLAST search (http://blast.ncbi.nlm.gov). These proteins were aligned with the program MUSCLE [31] using MEGA v7.0.25 software [32] and the maximum likelihood method based on the JTT matrix-based model [33].

### 2.4. Fibroblasts and Keratinocytes Viability and Proliferation Assays

Fibroblasts (3T3 lineage) and keratinocytes (HaCaT lineage) were culture in Dulbecco's modified Eagle's medium (DMEM, Sigma Aldrich, St. Louis, MO, USA), supplemented with 10% fetal bovine serum (Sigma Aldrich) and 0.2% gentamycin (Gilbco by Life Technologies). Cells were maintained in a controlled atmosphere, at 37°C with 10% CO_2_. In the cell viability assay, HaCaT and 3T3 cells were plated in 96 well plates at 10^4^ cells /well and incubated for 24 h at 37°C and 10% CO_2_. Then, cells were incubated with different concentrations of *L. m. muta* crude venom or its purified fraction (1.25–20 *μ*g/mL). After the incubation period, the culture supernatant was removed, and Alamar Blue (10% v/v) was added to each well, according to the protocol described by Damico [34], with modifications. The plate was incubated for 3 hours, and the produced fluorescence was measured using Synergy 2 (BioTek) at 540 nm for excitation and 590 nm for emission. For analysis of cell proliferation, cells were incubated with two different concentrations of LAPLA2 (10 and 20 *μ*g/mL) or crude venom for 24, 48, and 72 hours.

### 2.5. Animals and Ethics

Male Swiss–Webster mice (*n* = 36, 7 weeks old) weighing 25 ± 5 g were used in the model of excisional skin injury. Mice were allocated in individual cages cleaned daily, which were maintained under a controlled environment with 12 : 12 h light/dark conditions, 22 ± 2°C temperature, and 60–70% humidity. Rodent diet and water were provided *ad libitum*. All the procedures used in this study were approved by the Ethics Committee of Animal Experimentation of the Federal University of Viçosa (registration 365/2015).

### 2.6. Experimental Design

Prior to creating the wounds, animals were anesthetized by intraperitoneal injection of pentobarbital (70 mg/kg b.w.). Dorsolateral shaving of the animals was performed, and the area was degreased with ethyl ether (Merck, Rio de Janeiro, RJ, Brazil), followed by the use of 70% ethanol and 10% povidone-iodine for antisepsis (Johnson Diversey, Rio de Janeiro, RJ, Brazil). One circular wound with 10 mm in diameter was made with a scalpel in the skin until the dorsal muscular fascia was exposed [35]. Animals were randomly distributed in four groups (*n* = 9 per group): C (negative control): 0.9% NaCl solution; SS (positive control): 0.6 g of silver sulfadiazine (1%), L1 group: 5 *μ*L of LAPLA2 diluted in Milli *Q* water (100 *μ*L) (0.5%); and L2 group: 5 *μ*l of LAPLA2 diluted in Milli *Q* water (200 *μ*L) (0.25%). The concentration of the PAPLA2 was chosen based on the in vitro analysis. These concentrations showed best results for protecting the cells, showing decrease in the death of epithelial cells. All wounds were cleaned daily with 0.9% saline solution before each treatment. Wounds were topically treated daily for 12 days. Over this period, the time-dependent healing process was evaluated from the scar tissue collected every 4 days from three animals per group.

### 2.7. Wound Contraction Assay

The time-dependent wound closure was evaluated by measuring the wound area every 4 days in digitized images with the dimensions of 320 × 240 pixels (24 bits/pixel), obtained using a digital video camera (W320, Sony, Tokyo, Japan). The wound area was calculated by computerized planimetry using the software Image Pro-Plus, version 4.5 (Media Cybernetics, Silver Spring, USA), previously calibrated. The Wound Contraction Index (WCI) was calculated using the following ratio: initial area of the wound (Ao)−area measured on a given day (Ai)/initial area of the wound (Ao) × 100 [35]. The photos were standardized by the use of camera holder with standardized distance, and this procedure was performed for all the groups. The animals were sedated during this procedure.

### 2.8. Microstructural Analysis of Tissue Cellularity, Blood Vessels, Collagen Fibers, and Mast Cells

Skin sections were immersed in Karnovsky fixative for 24 h, dehydrated in ethanol, diaphanized in xylene, and embedded in paraffin. Histological sections with 5-*μ*m thickness were obtained using a rotary microtome (Leica Multicut 2045, Reichert- Jung Products, Jena, Germany) and stained with hematoxylin and eosin (H & E) for analysis of tissue cellularity and blood vessel [36]. Scar tissue sections stained with toluidine blue were used for mast cells identiﬁcation and quantification [37]. Sirius red (Sirius red F3B, Mobay Chemical Co., NJ, USA) was used for the collagen analysis. Collagen fibers were characterized and quantified from differential properties of birefringence under polarized light, in which thick collagen fibers (type I) appear in shades of bright colors ranging from red to yellow, whereas the thin reticular fibers (type III) appear bright green [38, 39].

Images were captured using a BX-60 bright-ﬁeld photomicroscope (Olympus, Tokyo, Japan). Stereological parameters of volumetric density (Vv) were calculated by counting the points over ﬁbroblasts, blood vessels, and type I and type III collagen, using the ratio Vv = PP/PT, in which PP is the number of points occurring over the structure of interest, and PT is the total number of points on the test system [40]. For this analysis, a grid containing 500 points within a standard test area (TA) of 73.10^3^ *μ*m^2^ at the tissue level was used. The number of mast cells per unit of histological area was calculated according to the notation QA = Σ mast cells/TA [41]. Ten histological ﬁelds were randomly sampled in each skin section using a 20x objective lens, and all microstructural parameters were analyzed in a total tissue area of 6.21 × 10^6^ *μ*m^2^. The Image Pro-Plus software (Media Cybernetics, Silver Spring, USA), was used in all quantifications [35].

### 2.9. Endogenous Antioxidant Enzymes Assay

Tissue fragments were collected from each wound, rapidly frozen in liquid nitrogen, and stored at −80°C. Samples were homogenized in phosphate buffered saline (PBS) and centrifuged at 5°C [42, 43]. The supernatant was used for the analysis of superoxide dismutase (SOD) and catalase (CAT). The SOD activity was estimated by a method based on the production of H_2_O_2_ by xanthine oxidase and reduction of nitroblue tetrazolium, according to the protocol described by Siddiqui [44]. The CAT activity was measured by measuring the rate of decomposition of hydrogen peroxide (H_2_O_2_) according to Aebi [45]. The enzymatic activity was normalized by the total protein levels in the supernatant, quantiﬁed according to the method described by Bradford [46].

### 2.10. Lipid and Protein Oxidation Assay

For the analysis of malondialdehyde (MDA), an end product of lipid peroxidation, the same supernatant was reacted with thiobarbituric acid, and the formation of thiobarbituric acid-reactive substance was monitored at 535 nm in accordance with the protocol described by Gutteridge and Halliwell [47]. Carbonyl proteins (CP), a direct marker of protein oxidation, were analyzed from the derivatization of the carbonyl group with 2, 4-dinitrophenylhydrazine (DNPH), which leads to the formation of a stable 2, 4-dinitrophenyl (DNP) hydrazone product. CP levels were measured by spectrophotometry at 370 nm according to the protocol described by Jana [48]. The results of MDA and CNP data were equally normalized by the total protein levels [46].

### 2.11. Statistical Analysis

Results are expressed as mean and standard deviation (mean ± SD). Comparisons among data with normal distribution were performed using one-way ANOVA followed by the Student–Newman–Keuls post hoc test. Nonparametric data were compared by the Kruskal–Wallis test. Statistical signiﬁcance was set at *P* < 0.05.

## 3. Results

### 3.1. LAPLA2 Isolated from Snake Venom

**Figure 1:**
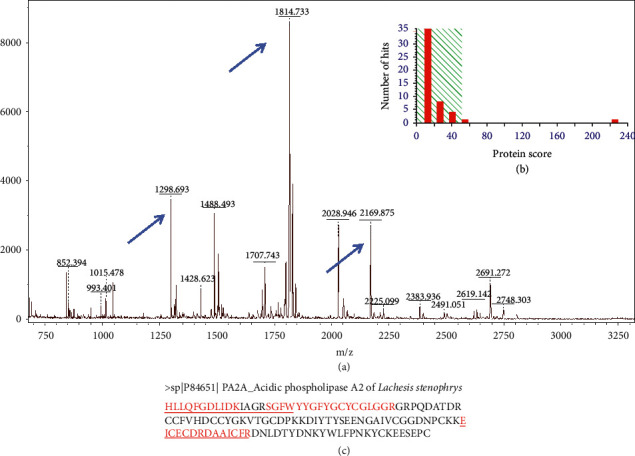
(a) MS1 of the L17 snake venom fraction, indicating in the blue arrows the peaks that followed for MS2 and matched with a protein in the database studied. (b) Mascot histogram with protein score represented by 216 bar. Individual ions scores >51 indicated identity or extensive homology (*P* < 0.05). (c) Tryptic peptides sequence identified and aligned in the complete sequence of PA2A of *L. stenophrys*.

LAPLA2 showed a band between ∼12 kDa and ∼17 kDa. After enzymatic digestion of the visualized protein, tryptic peptides were shown on the MS spectrum (Figure 1(a)). The obtained sequence represents ∼35% of the protein sequence (Figure 1(b)). The peptides m/*z* 1298.698 (HLLQFGDLIDK), m/*z* 1814.733 (EICECDRDAAICFR), and m/*z* 2169.875 (SGFWYYGFYGCYCGLGGR) were fragmented and aligned with the acidic phospholipase *A*_2_ (PA_2_A) sequence of *Lachesis stenophrys* (Figure 1(c)). The MASCOT result showed that LAPLA2 identification was highly reliable, with a score of 226, significance >51, and *P* < 0.05. The peptides were validated by SCAFFOLD software (Table S1). The BLAST analysis showed sequence homology with other acidic phospholipases of viperid snakes, and three conserved domains (PA2c, Phospholip_A2_1, and PLA2c) were identified. The alignment showed that the partial sequence of LAPLA2 was identical to a *Lachesis stenophrys* sequence, showing high homology (Figure S2).

### 3.2. Cell Viability and Proliferation In Vitro

**Figure 2:**
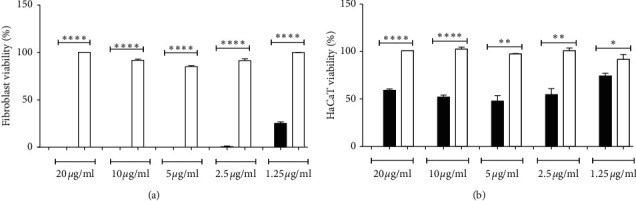
Effect of crude snake venom and isolated LAPLA2 on 3T3 fibroblast (a) and HaCaT keratinocytes (b) viability. Data are presented as mean ± standard deviation. Statistical differences ^*∗*^*P* < 0.05, ^*∗∗∗*^*P* < 0.001, and ^*∗∗∗∗*^*P* < 0.001.

**Figure 3:**
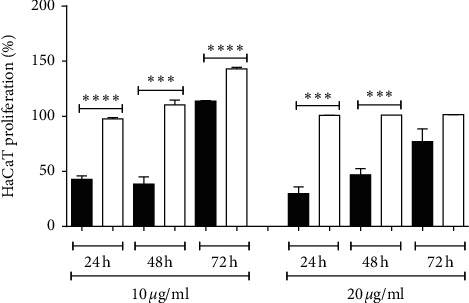
Effect of crude snake venom and isolated LAPLA2 on HaCaT keratinocytes proliferation. Data are presented as mean ± standard deviation. Statistical differences compared to the group (C) statistical difference ^*∗∗∗*^*P* < 0.001 and ^*∗∗∗∗*^*P* < 0.001.

The treatment of 3T3 fibroblasts with crude venom decreased cell viability (*P* < 0.01), whereas LAPLA2 did not affect cell viability (Figure 2(a)). In HaCaT keratinocytes, the highest concentration of crude venom (20 *μ*g/mL) induced a decrease (59%) in cell viability (*P* < 0.01), whereas HaCaT keratinocytes treated with LAPLA2 were 100% viable in all concentrations tested (Figure 2(b)). In relation to cell proliferation, with 10 *μ*g/mL of LAPLA2, increased keratinocytes proliferation was observed when compared to crude venom (*P* < 0.01). In contrast, the cells treated with LAPLA2 at 20 *μ*g/ml did not present a progressive increase in cellularity, but maintained the cell viability when compared to crude venom (*P* < 0.01) (Figure 3).

### 3.3. Wound Area and Contraction

All wounds were performed in aseptic condition, and no evidence of infection was detected. Time-dependent wound contraction was clearly observed in all groups of treatment. Wound area was significantly reduced in the L1 group in day 8 and day 12 (*P* < 0.01) compared with the C group. While the WCI was significantly higher in the L1 group, compared with the C group in days 8 and 12 (*P* < 0.05) (Table 1).

### 3.4. Tissue Cellularity and Vascularization in the Scar Tissue

**Figure 4:**
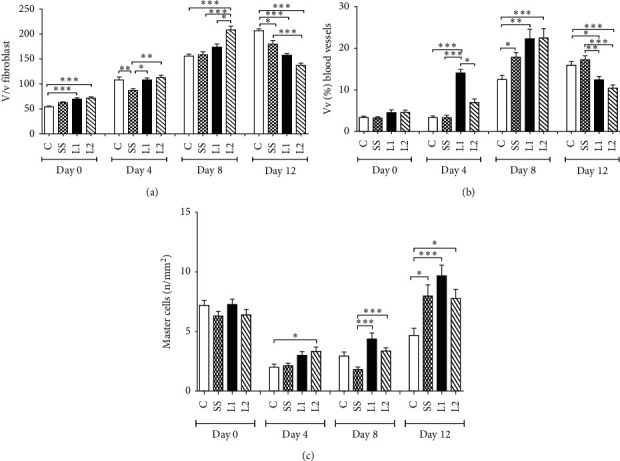
Proportion of fibroblasts (a), blood vessels (b), and mast cells (c) in the scar tissue of mice treated with LAPLA2 isolated from snake venom. C group: saline solution (0.9%), SS group: silver sulfadiazine (1%), L1 group: LAPLA2 (0.5%), and L2 group: LAPLA2 (0.25%). Data are presented as mean ± standard deviation. Statistical differences compared to the group (c) ^*∗*^*P* < 0.05, ^*∗∗*^*P* < 0.01, and ^*∗∗∗*^*P* < 0.001.

LAPLA2-treated animals showed an increase on tissue cellularity compared to the SS group on day 4. At day 8, the L2 group showed increased cellularity when compared to the other groups. On day 12, the two treatment groups showed decreased cellularity compared to the control (*P* < 0.001), and the L2 group showed decreased values compared to the SS group (*P* < 0.001) (Figure 4(a)).

**Figure 5:**
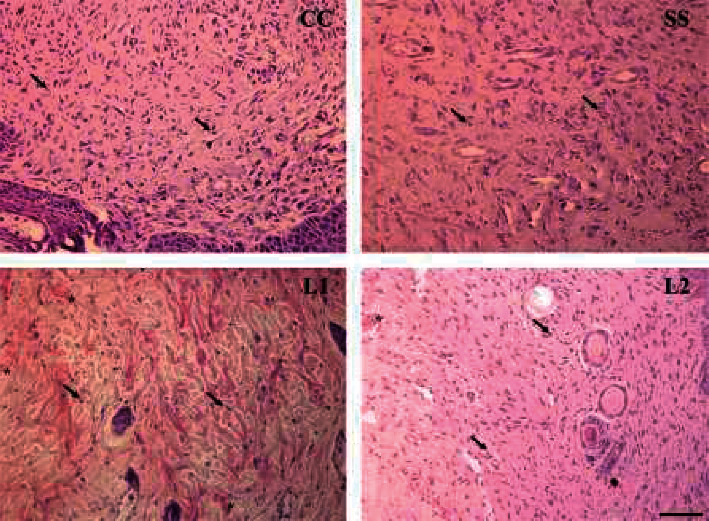
Representative photomicrographs of the scar tissue of mice treated with LAPLA2 isolated from snake venom. The images are representative of the scar tissue collected at day 12, indicating the most advanced healing process in all groups. C group: saline solution (0.9%), SS group: silver sulfadiazine (1%), L1 group: LAPLA2 (0.5%), and L2 group: LAPLA2 (0.25%). Tissue cellularity was more pronounced in the groups C and SS, while L1 and L2 animals exhibited marked distribution of cells (arrows) and vessels (asterisk). Bright field microscopy, hematoxylin and eosin staining, and scale bar: 20 *μ*m.

After 4 days, the L1 group showed an increase in blood vessel density compared to the groups control, SS, and L2 (*P* < 0.05). At day 8, blood vessel density increased in L1, L2, and SS groups compared to the control group (*P* < 0.05). On day 12, the density of blood vessels decreased in L1 and L2 groups compared to the groups control and SS (*P* < 0.05) (Figure 4(b)). In mast cells, there was a significant increase in the L2 group compared to the control group (*P* < 0.05) at the day four. L1 and L2 groups also increased significantly at day 8 compared to the SS group (*P* < 0.001). At day 12, the number of mast cells for SS, L1, and L2 groups remained high in the LAPLA2-treated groups when compared to the control (Figure 4(c)). The advanced healing process in the scar tissue of mice treated with LAPLA2 is represented in Figure 5.

### 3.5. Collagen Deposition in the Scar Tissue

**Figure 6:**
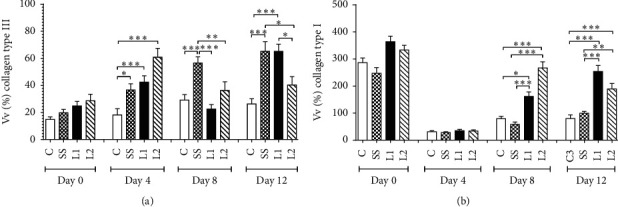
Proportion of type III (a) and type I (b) collagen fibers in the scar tissue of mice treated with LAPLA2 isolated from snake venom. C group: saline solution (0.9%), SS group: silver sulfadiazine (1%), L1 group: LAPLA2 (0.5%), and L2 group: LAPLA2 (0.25%). Data are presented as mean ± standard deviation. Statistical differences ^*∗*^*P* < 0.05, ^*∗∗*^*P* < 0.01, and ^*∗∗∗*^*P* < 0.001.

**Figure 7:**
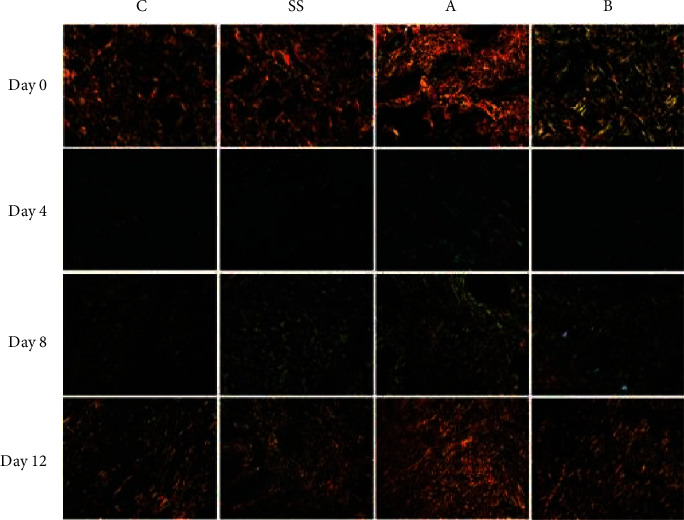
Representative photomicrographs obtained under polarizing microscopy demonstrate the distribution of collagen fibers in the scar tissue of rats treated with LAPLA2 isolated from snake venom. C group: saline solution (0.9%), SS group: silver sulfadiazine (1%), L1 group: LAPLA2 (0.5%), and L2 group: LAPLA2 (0.25%). Data are presented as mean ± standard deviation.

At day 4, type III collagen was higher in L1, L2, and SS groups when compared to control animals (*P* < 0.05). At day 8, a greater deposition of type III collagen was observed in the SS group. At day 12, the same parameter was increased in L1 and SS groups compared to the control group and L2 group (*P* < 0.05) (Figure 6(a)). At day 8, the L1 group showed an increase in type I collagen fibers deposition compared to control and SS groups (*P* < 0.05), while the L2 group presented higher values when compared to control and SS groups (*P* < 0.001). At day 12, type I collagen deposition was higher in the L1 group when compared to control and SS groups (*P* < 0.001). In the same day, the L2 group presented higher values than SS and control groups (*P* < 0.01) (Figure 6(b)). Control and SS groups showed a marked distribution of type III collagen fibers (green), while L1 and L2 groups presented a predominance of type I collagen fibers (yellow/red) (Figure 7).

### 3.6. Activity of Antioxidant Enzymes in the Scar Tissue

**Figure 8:**
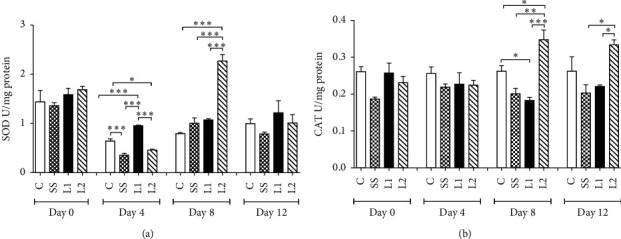
Time-dependent activity of superoxide dismutase (a) SOD and catalase (b) CAT in the scar tissue of mice treated with LAPLA2 isolated from snake venom. C group: saline solution (0.9%), SS group: silver sulfadiazine (1%), L1 group: LAPLA2 (0.5%), and L2 group: LAPLA2 (0.25%). Data are presented as mean ± standard deviation. Statistical differences ^*∗*^*P* < 0.05, ^*∗∗*^*P* < 0.01, and ^*∗∗∗*^*P* < 0.001.

The activity of SOD was higher at day 4 in the L1 group compared to L2, SS, and control groups (*P* < 0.001). The SOD activity was significantly higher in the control than SS and L2 animals (*P* < 0.05). At day 8, the L2 group showed a higher SOD activity when compared to the others groups (*P* < 0.001) (Figure 8(a)). The CAT activity increased at day 8 in the L2 group compared to L1, SS, and C groups (*P* < 0.05). The CAT activity was also higher in the control than that in L1 animals (*P* < 0.05). At day 12, CAT levels from the L2 group remained higher compared to SS and L1 groups (*P* < 0.05) (Figure 8(b)).

### 3.7. Lipid and Protein Oxidation in the Scar Tissue

**Figure 9:**
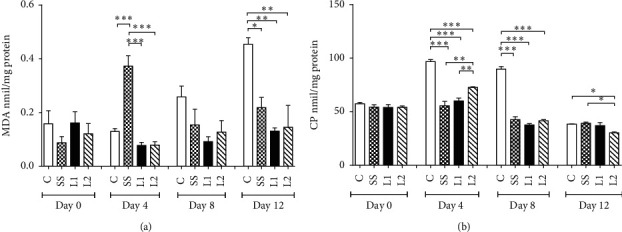
Malondialdehyde (a) MDA and carbonyl proteins and (b) CP in the scar tissue of mice treated with LAPLA2 isolated from snake venom. C group: saline solution (0.9%), SS group: silver sulfadiazine (1%), L1 group: LAPLA2 (0.5%), and L2 group: LAPLA2 (0.25%). Data are presented as mean ± standard deviation. Statistical differences ^*∗*^*P* < 0.05, ^*∗∗*^*P* < 0.01, and ^*∗∗∗*^*P* < 0.001.

At day 4, MDA levels were decreased in L1, L2, and control groups as compared to the group SS (*P* < 0.001). At day 12, MDA levels were decreased in SS, L1 and L2 groups as compared to that in control animals (*P* < 0.05) (Figure 9(a)).

The levels of carbonyl proteins were decreased in SS, L1, and L2 groups (*P* < 0.001) on days 4 and 8 compared to the control group. On day 12, CP levels were decreased in the L2 group compared to control and SS groups (*P* < 0.05) (Figure 9(b)).

## 4. Discussion

In the present study, we identified the healing effect of a phospholipase obtained from the venom of the snake *L. m. muta*. Our results showed that LAPLA2 does not affect the viability of fibroblasts and keratinocytes; on the contrary, it promotes cell proliferation. In addition, we found a decrease in wound area and an increase in cellularity and angiogenesis. The protein also stimulated the collagen deposition during the healing process and showed to have an antioxidant effect on the tissue. Furthermore, this work adds more information on the phospholipases found in the *Lachesis* genus by the identification of the new sequences and homologies.

Our results showed that LAPLA2 does not affect fibroblast and keratinocytes viability, indicating no evidence of toxicity in these integumentary lineages. Interestingly, LAPLA2 stimulated keratinocytes proliferation and modulated the morphofunctional dynamics of the scar tissue, accelerating wound contraction, and cutaneous repair. Another acid phospholipase isolated from the snake *Bothrops atrox* was shown to increase the inflammatory process, playing a role in the development of regional and systemic toxicity after exposure to venom [49]. On the other hand, PLA2s isolated from *B. asper* were also shown to be free of any toxic effect, although a slight edema was reported in treated animals, with no effects on the coagulation cascade or platelet aggregation [50]. Another acidic PLA2 isolated from *Porthidium lansbergii lansbergii* venom counteracted the toxic effects of the basic PLA2 isolated from the same snake [51].

Differently to the LAPLA2 effect in cell viability, our findings confirmed that *L. m. muta* crude venom was cytotoxic to fibroblasts, decreasing cells viability in culture. In addition, 50% reduction in HaCaT keratinocytes viability was clearly detected, indicating that these cells show partial resistance against all concentrations of venom used in the in vitro assays. This resistance was also observed in previous studies with HaCaT keratinocytes, which exhibited 70% cell viability under treatments at high doses (60 *μ*g/mL) of *Montivipera bornmuelleri* crude venom [52]. These findings corroborate the cytotoxic effect of this crude venom and the evidence that different cell populations of the integument have different resistance and susceptibility profiles to compounds present in snake venom [52, 53]. Therefore, it is prudent and advisable to investigate different cell types to evaluate the pharmacological potential and cytotoxicity risks associated with the different derivatives obtained from viperids' venom.

The proliferative stimulation of LAPLA2 on keratinocytes was consistent with our in vivo findings, indicating that this molecule was potentially effective in stimulating cell migration and proliferation in the scar tissue during the initial phases of wound healing (inflammatory and proliferative). Increased tissue cellularity is directly related with a marked inflammatory process and a better reepithelialization, which is potentiated when the wound closure is accelerated by healing products [54, 55]. Previous evidence indicates that proteins isolated from viperids' venoms are also able to induce a proliferative effect on retinal ganglion cells. Da Silva Cunha et al. [18] showed that the pharmacological potential of snake venoms derivatives is not restricted to integument cells. In addition to stimulating cell proliferation in the initial phases of wound healing, the effect of LAPLA2 extended to the later phase of tissue repair, contributing to the proper balance between cells and ECM components. In the remodeling stage, reduction of the inflammatory process and tissue cellularity, as well as intense colagenogenesis, are essential events, enhancing the strength and resistance of newly formed tissue [56–58]. Thus, by modulating both phases of wound healing, a faster wound closure in animas treated with LAPLA2 was expected, confirming its remarkable bioactive effect, with biotechnological potential in regenerative medicine.

Apart from fibroblasts and keratinocytes, mast cells also contribute to skin wound healing and are important effectors of the cutaneous immunological response [59, 60]. Although their role in the healing process is still not completely understood, studies have suggested that these cells have an implication in neovascularization by secreting stimulatory molecules such as cytokines (i.e., IL-1, TGF-*β*, and TNF-*γ*) and grown factors (i.e., FGF and VEGF) [61,62]. During the late phase of healing, a high number of mast cells on the edge of the wound stimulate degradation of thin collagen fibers (i.e., type III) by matrix metalloproteinases and colagenogenesis (especially mediated by FGF and TGF-*β*), playing a direct role in the reorganization of the fibrous matrix and morphofunctional remodeling of the skin [63, 64]. Mast cells also promote the recruitment of neutrophils after an injury, which play an important role on the scar tissue formation, and their lack is associated with delays in the healing process [65]. Our results show that LAPLA2 increased the distribution of mast cells in the wound edge, which is potently associated with an improved leucocytes recruitment, angiogenesis, colagenogenesis, and reorganization of collagen fibers, modulating the healing dynamics and the quality of tissue repair [64, 66].

Recent studies in vitro and in vivo also showed the proangiogenic activity on isolated proteins from viperids' venoms, which is effective in stimulating a better vascularization and nutrition potentially favorable for tissue repair [67, 68]. Although LAPLA2 has supported angiogenesis in the proliferative stage, progressive reduction in vascular density and tissue cellularity occurred simultaneously, and a process that is expected as the remodeling phase is established [69–71]. There is consistent evidence that after controlling the inflammation, the attenuation of trophic stimuli that occurs in response to lowering cytokine and growth factors levels activates the programmed death of a large cell population, especially leukocytes, fibroblasts, and endothelial cells [72, 73]. This event ensures that the secreted extracellular matrix is properly rearranged, approximating the morphofunctional characteristics of the repairing tissue to those typically observed in healthy skin [74].

In addition, collagenous proteins are essential for providing skin tensile strength and modulating cell migration in the scar tissue [75]. Due to intense colagenogenesis that occurs in the remodeling phase of wound healing, the marked increase in tensile strength of scar tissue occurs in response to the broad replacement of type III by type I collagen fibers [42, 76]. Similar to LAPLA2, the protein alternagin-C from *Bothrops alternatus* venom also is effective in stimulating colagenogenesis and scar tissue reorganization, increasing the deposition of type I collagen in rats' skin during wound healing [11]; in another work by Rabelo et al. [77], a disintegrin-like protein obtained from the venom of *B. alternatus* increased the collagen production in the fibrovascular tissue in mice. This effect was clearly observed in our study, since LAPLA2 from *L. m. muta* improved collagen maturation (type I/type II collagen ratio), which represents a direct morphological marker of wound closure.

Although the effects of LAPLA2 on skin biology is poorly understood, the modulation of oxidative processes in the scar tissue is potentially related with its healing properties observed in the present study. Animals treated with LAPLA2 showed an increased activity of the antioxidant enzymes SOD and CAT, which are important molecules involved in the modulation of tissue redox balance. Interestingly, this effect was beneficial in protecting the scar tissue against reactive tissue damage, since animals treated with LAPLA2 also exhibited decreased levels of lipid (i.e., MDA) and protein (i.e., CP) oxidation. The balance between the production of prooxidant molecules and antioxidant effectors is important for an efficient cutaneous repair [78]. When the tissue is damaged, the inflammatory process triggers an intense production of radical and nonradical prooxidant mediators (i.e., OH^•−^, O2^•−^, H_2_O_2_, and NO), which is directly associated to lipids, protein, and DNA oxidative damage that often culminates in disorganization of ECM and cell death [70, 79]. Through inducing H_2_O_2_ catalysis, increased SOD levels are shown to potentiate the skin healing process [80]. Due to an improved redox balance, CAT and SOD also contribute to create a molecular microenvironment favorable to stimulating fibroblast proliferation, migration, and activation, which culminate in adequate production and organization of ECM and a fast wound closure [81, 82]. Similar antioxidant effects were obtained with proteins purified from the venom of the snake *Naja naja*, which proved to be effective in preventing lipid peroxidation by stimulating SOD and CAT activities [83].

By improving the redox balance in injured tissues, LAPLA2 has demonstrated intriguing and relevant therapeutic potential, which deserves to be investigated in detail, since prolongation of the inflammatory process and oxidative tissue damage are partly responsible for the chronicity of skin lesions [84, 85].

The sequence of the monomeric acidic phospholipase isolated from *L. m. muta* venom is homologous to LsPA-1, an acidic phospholipase isolated from *L. stenophrys* [86]. According to the venom's biochemical characteristics, measured through the proteomic analysis, the sequence of *L. m. muta* is more closely related to *L. stenophrys* than to other species from the same genus [17], since both share the sequence SGFWYYG. Our work also contributes to the knowledge of phospholipases found in *Lachesis* genus, through the identification of the newly shared sequences HLLQFGDLIDK, EICECDRDAAICFR, and SGFWYYGFYGCYCGLGGR, which show high homology with LAPLA2 and LsPA-1 proteins [17].

## 5. Conclusion

Taken together, our findings indicate that snake venom may be a relevant source for the identification of molecules with pharmacological applicability in regenerative medicine. Thus, by inducing proliferative, proangiogenic, colagenogenic, and antioxidant effects, as well as stimulating the maturation of collagen fibers, the acidic phospholipase A2 isolated from *L. muta muta* venom was effective in accelerating the rate of wound closure in a murine excisional wound model. In addition to increasing the rate of skin healing, the administration of this phospholipase was safe, since it did not induce toxicity on fibroblasts and keratinocytes, integumentary cells essential for the repair of injured tissue from processes of reepithelization and reconstitution of the conjunctive matrix. Thus, LAPLA2 constitutes a promising and interesting molecule potentially useful in regenerative medicine to potentiate skin wound healing.

## Figures and Tables

**Table 1:** Wound area (mm^2^) and the Wound Contraction Index (WCI) (%) in animals treated with different concentrations of LAPLA2.

Day	Area/Contraction	C	SS	L1 0.5%	L2 0.25%
0	Area (mm^2^)	110.00 ± 0.00	100.00 ± 0.00	105.00 ± 0.00	108.00 ± 0.00
	WCI (%)	00.00 ± 00.00	00.00 ± 00.00	00.00 ± 00.00	00.00 ± 00.00

4	Area (mm^2^)	76.66 ± 9.42	83.33 ± 9.42	60.00 ± 21.60	80.00 ± 8.16
	WCI (%)	30.30 ± 10.49	16.66 ± 11.54	42.85 ± 25.19	25.92 ± 9.25

8	Area (mm^2^)	70.00 ± 16.32	53.33 ± 12.47	23.33 ± 4.71^*∗∗*^	46.66 ± 12.47
	WCI (%)	36.36 ± 18.1	46.66 ± 15.27	77.77 ± 5.49^*∗*^	56.79 ± 14.14

12	Area (mm^2^)	20.00 ± 00.00	13.33 ± 4.71	6.66 ± 4.71^*∗∗*^	11.66 ± 2.35
	WCI (%)	81.81 ± 00.00	86.66 ± 5.77	93.65 ± 5.49^*∗*^	89.19 ± 2.67

C group: saline solution (0.9%), SS group: silver sulfadiazine (1%), L1 group: LAPLA2 (0.05%), L2 group: LAPLA2 (0.25%). Data are presented as mean ± SD. Statistical differences compared to the group C, ^*∗*^*P* < 0.05 and ^*∗∗*^*P* < 0.01.

## Data Availability

The data used to support the findings of this study are included within the article.

## Supplementary Materials

Supplementary Materials Table S1: peptides (HLLQFGDLIDK), (EICECDRDAAICFR), and (SGFWYYGFYGCYCGLGGR) were fragmented and aligned with the acidic phospholipase A2 (PA2A) sequence of *Lachesisstenophrys*. Figure S1: multiple alignment of LAPLA2 partial amino acid sequences with five homologous phospholipases A2 sequence from snake venoms. The UniProtKB access codes and the species for the acidic enzyme sequences are shown in boldfaces. Sequences are aligned by MUSCLE in MEGA v7.025 software. Identical amino acid positions are in yellow.
