# Supplementary material for: Acidic Phospholipase A2-Peptide Derivative Modulates Oxidative Status and Microstructural Reorganization of Scar Tissue after Cutaneous Injury
Source: Evid Based Complement Alternat Med. 2020 Jul 11;2020:8273986. doi: 10.1155/2020/8273986 (PMC7369679; doi:10.1155/2020/8273986)

**Fig. S1.** Multiple alignment of LAPLA_2_ partial amino acid sequences with five homologous phospholipases A2 sequence from snake venoms. The UniProtKB access codes and the species for the acidic enzyme sequences are shown in boldfaces. Sequences aligned by MUSCLE in MEGA v7.025 software. Identical amino acid positions are in yellow.


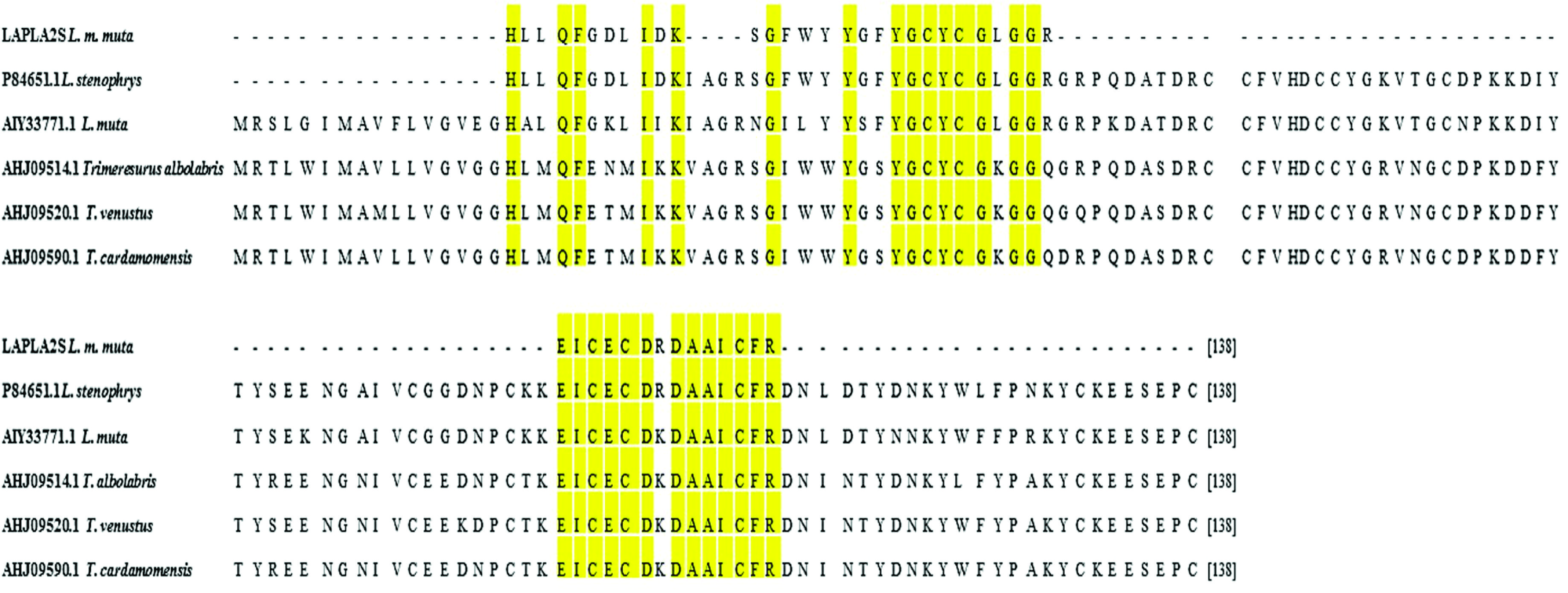

Supplement: Supplementary Materials — Table S1: peptides (HLLQFGDLIDK), (EICECDRDAAICFR), and (SGFWYYGFYGCYCGLGGR) were fragmented and aligned with the acidic phospholipase A2 (PA2A) sequence of Lachesisstenophrys. Figure S1: multiple alignment of LAPLA2 partial amino acid sequences with five homologous phospholipases A2 sequence from snake venoms. The UniProtKB access codes and the species for the acidic enzyme sequences are shown in boldfaces. Sequences are aligned by MUSCLE in MEGA v7.025 software. Identical amino acid positions are in yellow. [file 8273986.f1.zip › 8273986.f1/Figure S1.docx]
